# A Nitrite Biosensor Based on Co-immobilization of Nitrite Reductase and Viologen-modified Chitosan on a Glassy Carbon Electrode

**DOI:** 10.3390/s100606241

**Published:** 2010-06-22

**Authors:** De Quan, Woonsup Shin

**Affiliations:** 1 Department of Chemistry and Interdisciplinary Program of Integrated Biotechnology, Sogang University, Seoul, 121-742, Korea; 2 Department of Chemistry, College of Chemistry, Chemical Engineering and Environment, Qingdao University, Qingdao, Shandong, 266071, China

**Keywords:** biosensor, nitrite reductase, chitosan, viologen, hydrophilic polyurethane

## Abstract

An electrochemical nitrite biosensor based on co-immobilization of copper- containing nitrite reductase (Cu-NiR, from *Rhodopseudomonas sphaeroides* forma sp. *denitrificans*) and viologen-modified chitosan (CHIT-V) on a glassy carbon electrode (GCE) is presented. Electron transfer (ET) between a conventional GCE and immobilized Cu-NiR was mediated by the co-immobilized CHIT-V. Redox-active viologen was covalently linked to a chitosan backbone, and the thus produced CHIT-V was co-immobilized with Cu-NiR on the GCE surface by drop-coating of hydrophilic polyurethane (HPU). The electrode responded to nitrite with a limit of detection (LOD) of 40 nM (*S/N* = 3). The sensitivity, linear response range, and response time (*t*_90%_) were 14.9 nA/μM, 0.04−11 μM (*r*^2^ = 0.999) and 15 s, respectively. The corresponding Lineweaver-Burk plot showed that the apparent Michaelis-Menten constant (*K*_M_^app^) was 65 μM. Storage stability of the biosensor (retaining 80% of initial activity) was 65 days under ambient air and room temperature storage conditions. Reproducibility of the sensor showed a relative standard deviation (RSD) of 2.8% (n = 5) for detection of 1 μM of nitrite. An interference study showed that anions commonlyfound in water samples such as chlorate, chloride, sulfate and sulfite did not interfere with the nitrite detection. However, nitrate interfered with a relative sensitivity of 64% and this interference effect was due to the intrinsic character of the NiR employed in this study.

## 1. Introduction

Nitrite is a typical inorganic pollutant in environmental, food, industrial and physiological systems [1–3], therefore, various methods for detection and determination of nitrite have been reported, involving spectroscopic, chromatographic, electrochemical detection methods, *etc.* [3–5]. Although spectroscopic and chromatographic methods are accurate and reliable, they require bulky, delicate and expensive equipment which is not suitable for *in situ* applications. Biosensor-based methods have been another option for this purpose, for example, nitrate reductase (NiR)-based optical [6–8] and electrochemical [9–19] biosensors have been reported. In electrochemical detection, although direct electron transfers (DET) between electrode and NiRs were seldom reported [11,20,21], most of the reported electrochemical nitrite biosensors were based on co-immobilization of NiR and respective mediators. There have been various reported co-immobilization methods for NiR and mediators, such as electropolymerization [13,15], dialysis membrane [12,14] or polymer membrane covering [17], polymer entrapment [11], covalent binding on self-assembled monolayer (SAM) surface [16], electrostatic interaction [18], adsorption and following cross-linking [19], *etc.*, each of which have their respective merits and defects. In this study, a synthesized polymeric CHIT-V mediator and NiR were co-immobilized by drop-coating of HPU, due to its simplicity and effectiveness [22].

Recently, studies on structures and catalytic mechanisms of NiRs have been actively carried out [23–33]. NiRs can be classified into copper-based NiRs and iron-based NiRs. The iron-based NiRs include *cc*-NiRs which contain several c-type hemes or siroheme catalyzing 6-electron reduction of nitrite to ammonia and cytochrome *cd*_1_ NiR catalyzing 1-electron reduction of nitrite to nitric oxide. The Cu-NiRs are also known to catalyze 1-electron reduction reactions [12,26,29,30]. In the typical homotrimeric structures of Cu-NiRs, each monomer contains one type 1 copper (T1 Cu, ET site) which accepts one electron from an external electron carrier and one type 2 copper (T2 Cu, binding site of nitrite) which accepts an electron from the reduced T1 Cu [23–26,29,30]. T1 Cu is located 7 Å beneath a hydrophobic surface patch which is surrounded by many negatively charged residues [30,33] and T2 Cu is located at the bottom of a 12 Å [23] or 16 Å [34,35] deep cavity formed at the interface between two adjacent monomers, and the inter-atomic distance between the two Cu sites is 12.6 Å [30]. The NiR used in this study belongs to the copper-containing type from *Rhodopseudomonas sphaeroides* forma sp. *denitrificans*, which catalyzes 1-electron/2-proton reduction of nitrite to nitric oxide. This enzyme has a homodimeric structure (2 × 39 kDa) and contains both T1 and T2 Cu centers per monomeric unit [36]. The stability of this type NiR was reported to be good [36,37], which is essential to the preparation of a biosensor.

The redox potentials of T1 Cu of some Cu-NiRs are in the range of 0.04–0.1 V *vs.* Ag/AgCl at pH 7.0 [38], and that of Cu-NiR from *Rhodobacter sphaeroides* strain 2.4.3 is 0.05 V *vs.* Ag/AgCl at pH 7.0 [28]. Several ET mediators have been tested to electrochemically reduce the T1 Cu of Cu-NiRs [12], and methyl viologen has been proven to be useful as mediator/Cu-NiR systems for catalytic reduction of nitrite [17]. In this study, viologen was covalently linked to a chitosan backbone to effectively immobilize this mediator on the electrode surface by drop-coating of a HPU membrane. Chitosan is a natural biopolymer, and its unique properties such as biocompatibility, remarkable affinity for proteins, antibacterial property and environmentally friendly polyelectrolyte nature, *etc.* render this material suitable for biofabrication [39]. Chitosan has been functionalized to be applied in catalysis [40], has been used to immobilizations of more than 60 enzymes [41,42]. In addition, it was reported that chitosan is able to increase the stability and activity of immobilized enzymes [43]. Undoubtedly, these points are important for a biosensor to be used practically.

**Figure 1.**
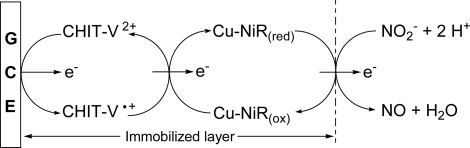
Scheme of working principle of the co-immobilized Cu-NiR and CHIT-V GCE.

In this paper, we report the electrochemical characterization of a co-immobilized Cu-NiR and CHIT-V GCE as a reagentless biosensor for nitrite detection. The working principle of the proposed biosensor is shown in Figure 1. This kind of scheme is common in mediated catalytic reactions [44].

## 2. Experimental

### 2.1. Chemicals and Materials

Sodium hydrogen phosphate (99.95%), sodium dihydrogen phosphate (99.999%), potassium chloride (99.999%), sodium chlorate (≥99.0%), sodium sulfite (≥98%), sodium sulfate (99.99+%), sodium nitrate (≥99.0%), sodium nitrite (99.999%), 4,4′-bipyridyl (99%), methyl iodide (99%), 3-bromopropylamine hydrobromide (99%), glutaraldehyde (GA, ∼25% in water), sodium cyanoborohydride (95%), chitosan (medium molecular weight (190,000–310,000), 75–85% deacetylation), chloroform (99.95%), acetone (AR Grade), methanol (AR Grade) and acetonitrile (AR Grade) were purchased from Sigma-Aldrich Korea and used as received. HydroThane™: hydrophilic thermoplastic polyurethane (HPU, aliphatic type (AL-25-80A), 25% water uptake) was purchased from CardioTech International, Inc. (Wilmington, MA, USA). All of other chemicals were of at least reagent grade and were used without further purification. Deionized water (18 MΩ·cm) from Milli Q water purification system was used for preparing buffer and stock solutions. Sodium nitrite solution was freshly prepared just before the experiment.

The lyophilized powder of nitrite reductase (EC 1.7.2.1, purified from *Rhodopseudomonas sphaeroides* forma sp. *denitrificans*) was purchased from NECi (Lake Linden, MI, USA), and used as received. The molecular mass of the enzyme is ∼80 kDa [36], and specific activity is 2 U/mg.

### 2.2. Synthesis of CHIT-V

**Figure 2.**
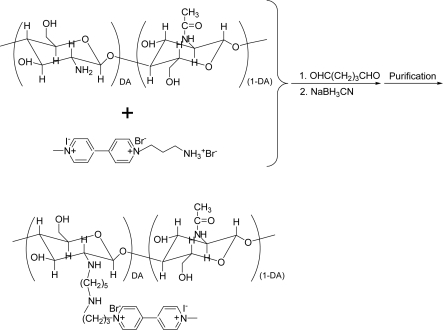
CHIT-V synthesis scheme.

1-(3-Ammoniopropyl)-1′-methyl-4,4′-bipyridinium (I^−^, Br^−^) (viologen, **1**) was synthesized according to the method we reported elsewhere [22] with some modifications. One wt% of chitosan solution (0.2 mL), which was prepared according to the reported procedures [45,46], was put into a flat-bottomed glass vial, and 0.7 mL of water was added. Into this chitosan solution 50 mg of viologen (**1**) was added, and the pH of the solution was slowly adjusted with dilute NaOH to ∼7.0. Then 0.1 mL of 2.5% of glutaraldehyde was added dropwise, and once again, the pH of this solution was adjusted to ∼7.0. The mixture was allowed to react with stirring for 4 hours at 60 °C. After the reaction the formed Schiff’s base was reduced by adding 1 mL of 0.5 M sodium cyanoborohydride for 1 hour at room temperature. The reaction mixture was transferred to a microcon (Utracel YM-3, 3000 MWCO) and the small molecules were removed by the ultrafiltration at 10,000 rpm. This operation was repeated six times by filling the tube with pure water until the filtrate did not show any detectable CV peaks for viologen species. After the separation of redox-active small molecules, the supernatant in the tube was collected as the target product, while the solid in the tube was discarded as by-product. The volume and concentration of obtained product were 150 μL and 0.3 wt%, respectively. The CHIT-V synthesis method is shown schematically in Figure 2.

### 2.3. Co-immobilization of NiR and CHIT-V

GCEs were polished successively with 2, 1 and 0.3 μm polishing powder (alumina, Buehler) on a polishing pad (Buehler), followed by sonication for 5 min in a mixed solution of water and ethanol (50 v/v %), and dried with nitrogen gas. Then, the required volumes of enzyme solution (3 mg/mL, in 0.05 M phosphate buffer (PB), pH 7.0) and CHIT-V solution (0.3 wt%) were thoroughly mixed in a 0.2 mL Eppendorf tube by vigorous vortexing. Three μL of the mixture was dropped on the pretreated GCE surface and allowed to dry in a vacuum dessicator at room temperature for 3 min. Typically, 3 μL of the mixture consisting of 1 μL of enzyme and 2 μL of CHIT-V was dropped on the GCE surface. On the top of the enzyme and CHIT-V loaded GCE surface 5 μL of 15 mg/mL HPU in chloroform was dropped carefully to cover the electrode with the HPU membrane. For the electrochemical characterization of the mediator 3 μL of CHIT-V was immobilized and similarly covered by HPU on the GCE but without enzyme.

### 2.4. Apparatus

An Electrochemical Analyzer (CHI 1230A, TX, USA) was used to carry out cyclic voltammetric and amperometric measurements. A conventional three electrode system consisting of a gassy carbon working electrode (3 mm diameter, BAS), platinum wire counter electrode (coil), and Ag/AgCl reference electrode (3 M KCl, BAS) was used. For amperometric measurements 5 mL of buffer solution in the closed cell was continuously stirred by a magnetic stirring bar. The applied potential (E_app_) was selected to be ∼0.1 V more negative than the cathodic peak potential (E_p,c_) of immobilized CHIT-V. Oxygen was thoroughly driven out from solution by purging with ultra pure argon gas (99.9999%, DongA Co, Korea) and experiments were all carried out under an atmosphere of argon. Experimental temperature was controlled to 25 ± 0.2 °C by use of a thermostated cell in all experiments. All potentials in the text are referred to Ag/AgCl (3 M KCl) reference electrode.

## 3. Results and Discussion

### 3.1. Synthesis of CHIT-V

CHIT-V was prepared by covalent linking of 1-(3-ammoniopropyl)-1′-methyl-4,4′-bipyridinium (I^−^, Br^−^) (**1**) to chitosan by use of glutaraldehyde. This crosslinker has been extensively used in linking of chitosan to other materials that bear amine functional groups [40,45]. Although a higher pH was favorable, the pH of the reaction mixture was adjusted to ∼7.0 because the solubility transition point of chitosan is ∼6.3 [39–42]. Reaction at this pH and 60 °C could greatly shorten the reaction time to ∼4 hours compared with that reported, typically 48 hours [46]. After the reaction, remaining small molecules (viologen) were separated from the target product by ultrafiltration. Subsequent electrochemical experiments confirmed that no viologen species remained in the filtrate. In addition, another CHIT-GA-CHIT by-product could be easily separated as a solid from the target product (a liquid). Chitosan used in this study has a high molecular weight (190,000–310,000), therefore, the dimer of chitosan has a rather limited solubility in the reaction mixtures, allowing us to collect viologen (**1**)-free CHIT-V.

### 3.2. Electrochemical Characteristics of Immobilized CHIT-V

**Figure 3.**
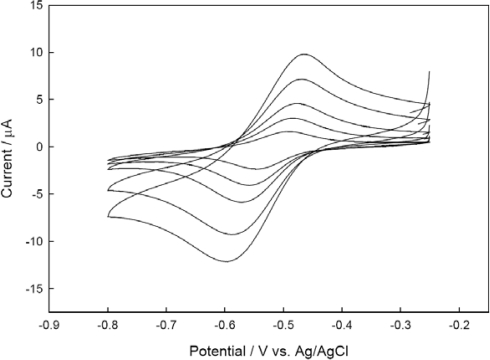
CVs recorded with CHIT-V immobilized GCE in 0.1 M phosphate buffer, pH 7.0 under argon. Scan rates are 0.002, 0.005, 0.01, 0.025 and 0.05 V/s, respectively in the direction of peak current increases.

**Figure 4.**
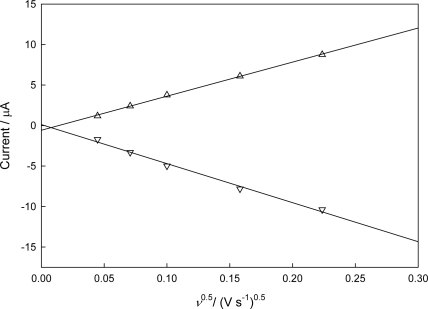
*i*_p_–*ν* profile of CHIT-V immobilized GCE (triangle down: *i*_p,c_; up: *i*_p,a_).

Figure 3 shows CVs recorded with 3 μL of CHIT-V immobilized on a GCE at various scan rates (*ν*) in 0.1 M PB, pH 7.0 under argon. As shown in Figure 4, both the cathodic peak current (*i*_p,c_) and the anodic peak current (*i*_p,a_) show linearity (*r*^2^ = 0.995 for *i*_p,c_ and *r*^2^ = 0.998 for *i*_p,a_) with square root of scan rate to 0.05 V/s, which is typical for a diffusion-limited case. Contrary to the case of a redox material directly immobilized on an electrode surface by adsorption or covalent binding, in which peak currents of immobilized redox material show linear behavior with the first power of the scan rate, the CHIT-V in this case was confined in a small space between the electrode surface and the swelled HPU membrane. Therefore, CHIT-V was free to move close to the electrode surface to give a diffusion-limited behavior. The peak separation (ΔE_p_) increases with the increase of scan rate, which can also be explained by the slow mobility of CHIT-V within the confined space since the redox active viologen species was covalently linked to the high molecular weight chitosan backbone. These phenomena were also observed in a similar case where viologen-modified polysiloxane (PAPS-V) was immobilized on a GCE, also by drop-coating of HPU [22].

The obtained electrochemical characteristics of the immobilized CHIT-V at a low scan rate of 0.002 V/s are as follows: ΔE_p_ = 0.056 V, *i*_p,a_/*i*_p,c_ = 0.696 and E^0′^ = −0.518 V. The charge of the cathodic wave measured by integration at 0.002 V/s is 8.0 × 10^−5^ C, which corresponds to 8.3 × 10^−10^ moles of viologen species on the GCE surface, in other words, surface coverage (Γ_0_^*^) is 1.2 × 10^−8^ mol cm^−2^. Since normally 2 μL of 0.3 wt% solution of CHIT-V was loaded on the electrode surface, the amount of monomeric unit containing one terminal amine group on the surface can be calculated to be 3.7 × 10^−8^ mol. This leads to the conclusion that only one of every 55 chitosan amines was linked to viologen by assuming the molecular weight of the chitosan and degree of deacetylation be 250,000 g mol^−1^ and 80%, respectively.

### 3.3. Catalytic Behavior of the Co-immobilized NiR and CHIT-V GCE

**Figure 5.**
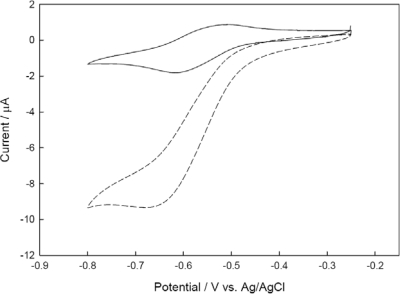
CVs recorded with NiR and CHIT-V co-immobilized GCE in 0.1 M phosphate buffer, pH 6.5 under argon in absence (solid line) and presence (dash line) of 2 × 10^−3^ M nitrite. Scan rates: 0.002 V/s.

Before the check of the catalytic behavior of the co-immobilized NiR and CHIT-V GCE, stabilities of the co-immobilized elements were tested (data not shown). Stable successive CV scans showing ΔE_p_ = 0.159 V and *i*_p,a/_*i*_p,c_ = 0.657 were obtained. A similar result was also obtained in the case where NiR and PAPS-V were co-immobilized on a GCE by drop-coating of HPU [22]. Figure 5 shows CVs recorded with the co-immobilized NiR and CHIT-V GCE at a scan rate of 0.002 V/s.

As shown in this figure, after addition of nitrite the reduction current increased greatly accompanied by the disappearance of the oxidation current. This is a typical EC’ process [47] and the sigmoid shape of the CV corresponds to the mediated catalytic reduction scheme shown in Figure 1. Similar electro-enzymatic catalytic reductions of nitrite by NiR and poly-viologen co-immobilized electrodes were also reported [15,22]. Control experiments were also conducted under similar conditions with a co-immobilized CHIT-V and bovine serum albumin (BSA) electrode (no NiR, data not shown), and those results showed that no catalytic reduction current could be observed, as reported [15,17–19].

### 3.4. Optimization of the Co-immobilized NiR and CHIT-V GCE

**Figure 6.**
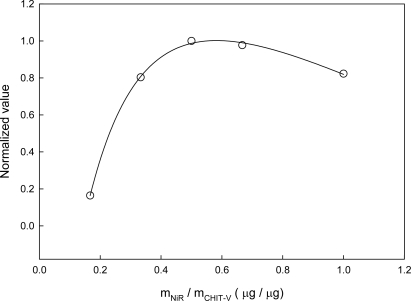
Effect of relative amount of co-immobilized NiR and CHIT-V (m_NiR_:m_CHIT-V_) on catalytic reduction of nitrite. Catalytic reduction currents for 1 mM nitrite at scan arte of 0.002 V/s were compared. The normalized value was calculated relative to the maximum value of the catalytic current.

The co-immobilized NiR and CHIT-V GCE was optimized. Undoubtedly, the response of the electrode should be directly dependent on the relative amount of immobilized NiR and CHIT-V. As shown in Figure 6, the electrode response increases sharply with increases of the m_NiR_:m_CHIT-V_ mass ratio to a maximum at a ratio of 1:2 (3 μg:6 μg), followed by slow decreases above this ratio, showing an unsymmetrical bell-shape.

**Figure 7.**
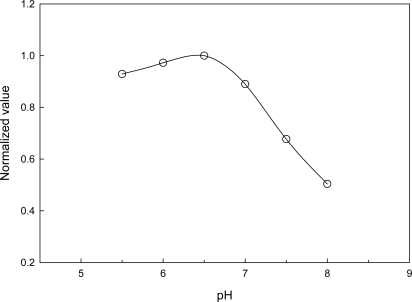
pH profile of NiR and CHIT-V co-immobilized GCE in 0.1 M phosphate buffer under argon. Catalytic reduction currents for 1 × 10^−3^ M of nitrite at scan rate of 0.002 V/s were compared.

This trend is typical of co-immobilized enzyme and mediator systems, and can be attributed to the balance among catalytic power of immobilized NiR, ET mediation efficiency of CHIT-V and mass transport limitation. A similar profile was also obtained with NiR and polyviologen co-immobilized on a GCE [15], and in another similar study with NiR and PAPS-V co-immobilized on a GCE, a mass ratio of 1:1 (2.5 μg:2.5 μg) was obtained [22]. In the case reported here, 6 μg of CHIT-V resulted in immobilization of 8.3 × 10^−10^ mol of viologen (see discussion in Section 3.2), which equals 9.2 × 10^−11^ mol viologen·μg^−1^ CHIT-V. Similarly, a value of 2.3 × 10^−10^ mol viologen·μg^−1^ PAPS-V was obtained for the reported case [22]. Very interestingly, the optimal mass ratio obtained in this study is in good accordance with that reported in the other one, *i.e.,* 3.0 μg NiR:5.5 × 10^−10^ mol viologen (this study) *vs.* 2.5 μg NiR:5.8 × 10^−10^ mol viologen (reported). As mentioned in the Introduction, the overall reduction of nitrite to nitric oxide by Cu-NiR is a 1-electron/2-proton process and the enzyme stability is also pH dependent. Thus, pH would directly affect the response of the electrode. The relative activity profile is shown in Figure 7 and it shows a pH optimum of 6.5.

The sensor response decreases a little with decreasing pH in the 5.5−6.5 range. This pH profile is in accordance with that we reported previously [17,22], and this optimal pH is also very comparable to those for free Cu-NiRs purified from different sources, generally at pH 6.0−6.2 [25,30,31]. On the other hand, it was reported that the solubility transition point of chitosan is ∼6.3 [39–42], and this may be partially responsible for the sharp decreases of response in the 6.5−8.0 pH range. In this pH range chitosan gradually became insoluble with increasing pH and unfavorable for the interaction with NiR for intermolecular electron transfer. In addition, the pH-sensitive swelling of chitosan hydrogel was also reported [48]. Obviously, swelling of the chitosan network at low pH would be advantageous for mass transport and intermolecular ET. The optimal pH for immobilized NiR shown here is similar to those for free Cu-NiRs, which implies that the immobilization method employed in this study caused almost no configuration change for the enzyme in the immobilization process.

We also tested effect of buffer concentration on sensor response (data not shown), and results showed that the highest reduction current corresponds to 0.1 M in the tested range of 0.05−0.4 M. In addition, the optimal HPU concentration in the immobilization process was selected to be 15 mg/mL (in chloroform) based on the previous result [22].

### 3.5. Performance Factors of Co-immobilized NiR and CHIT-V on GCE as a Biosensor

**Figure 8.**
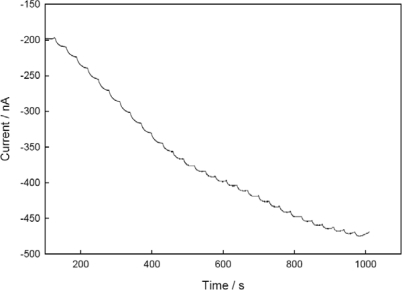
Steady-state current response of NiR and CHIT-V co-immobilized GCE to successive nitrite injections in 0.1 M phosphate buffer, pH 6.5 under argon. E_app_= −0.75 V, 1.0 μM of nitrite per injection.

Figure 8 shows the typical steady-state current response of the sensor to successive nitrite concentration increments (1 μM) under optimal conditions. Substrate concentrations were changed by stepwise addition of a concentrated solution to a stirred buffer. As shown, the obtained amperometric signal at the low concentration of nitrite is stable and clear, which may originate from the stable sensing system on the electrode surface and efficient ET between the electrode and immobilized enzyme. The response time (*t*_90%_) of the sensor was ∼15 s, which is considerably faster than that of other membrane-based biosensors (*t*_95%_ = 3 min) [12], and relatively faster than that of the co-immobilized NiR and PAPS-V sensor (*t*_90%_ = 60 s) [22]. The fast response is one of the merits of the biosensor, which may be partially ascribed to the higher degree of porous structure of chitosan backbone [48].

**Figure 9.**
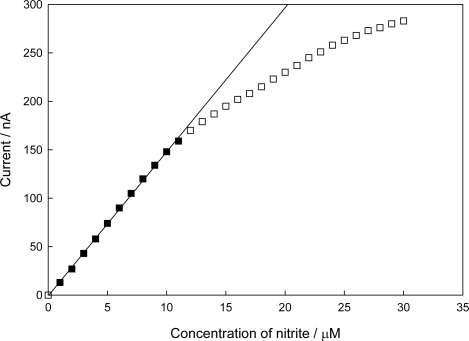
Calibration curve of the biosensor.

Figure 9 shows the calibration curve of the sensor at low nitrite concentration. The sensitivity, linear detection range and LOD of the sensor were 15 nA/μM, 0.04−11 μM (*r*^2^ = 0.999) and 40 nM (*S/N* = 3), respectively. These performance factors of the sensor are comparable to those reported in the literature [12–19], and variations in these factors may be due to the different substrate specificities of NiRs from different sources, different electrode materials, different mediators and different immobilization methods, *etc.*

The obtained sensitivity can be estimated to be 0.21 μA/μM·cm^2^ (0.3 cm in diameter), and this value is only one ninth of that obtained by heme-NiR immobilized sensor (1.8 μA/μM·cm^2^) [19]. It is noteworthy that reduction of nitrite by *cc*-NiR is a 6-electron process, whereas that by Cu-NiRs is a 1-electron process. Taking into account the different catalytic mechanisms of the two NiRs, the sensitivity obtained in this study is then comparable to the reported one.

Obviously, the high sensitivity of the sensor reported here originated from the efficient ET between electrode and immobilized NiR (see also discussion on *K*_M_^app^). The obtained linear detection range fully covers the nitrite concentration range encountered in water samples, for example, the acceptable nitrite maximum in drinking water is ∼2.2 μM for the EU standard [7–9,15]. Deviation from linearity at higher concentration of nitrite was observed, which is a typical phenomenon for an enzyme electrode, and can be ascribed partially to the saturation of the immobilized NiR, or inhibition of the immobilized NiR by the enzymatic nitric oxide product [26]. The obtained LOD is much lower than the standard value, as mentioned, making this sensor applicable in practical detections.

In our kinetic study, the Lineweaver-Burk plot based on Figure 9 (data not shown) indicates that the apparent Michaelis-Menten constant (*K*_M_^app^) was 65 μM. This value is 43% of that obtained by a Cu-NiR (from *Rhodobacter sphaeroides* strain 2.4.3) immobilized nitrite biosensor (*K*_M_^app^ = 150 μM) [13], and 65% of that obtained by a co-immobilized Cu-NiR (the same enzyme used here) and PAPS-V sensor (*K*_M_^app^ = 101 μM) [22]. The lower *K*_M_^app^ value obtained in this study means that mass transport of ions through HPU membrane and NiR/CHIT-V composite network was relatively easy and without many limitations. The higher degree of porous structure of the chitosan backbone [48] may be responsible for it. The lower *K*_M_^app^ value also means that CHIT-V has a higher affinity for co-immobilized Cu-NiR. As mentioned in the Introduction, the T1 Cu site of NiR is located 7Å beneath a hydrophobic surface patch which is surrounded by many negatively charged residues [30,33], therefore, viologen moieties linked to chitosan backbone could favorably interact with T1 Cu site of NiR by electrostatic and hydrophobic interactions. Similarly, it was already pointed out that the electrostatic interaction controls the rate of intermolecular ET reaction between Cu-NiR and a small pseudoazurin protein [33]. In addition, some other interactions such as hydrogen bonding, salt bridge (or polar interaction) and hydrophilic contact may be also involved in the favorable interaction of CHIT-V with Cu-NiR because chitosan has some unique characteristics such as biocompatibility and remarkable affinity for proteins [39–42]. Similarly, Cosnier *et al*. pointed out that hydrophobic interactions between heme-NiR and polypyrrole may enhance the protein structure stability and hence counterbalance the matrix denaturation effects [15].

RSD of the sensor was 2.8% for those prepared in the same set (n = 5). This value is comparable to those obtained with Cu-NiR immobilized nitrite biosensors for which the RSD values were 3.0% (n = 6) [13] and 3.2% (n = 5) [22], respectively. However, the RSD of the sensor prepared in different sets (n = 10) was relatively high at 6.0%, which may be mainly attributed to variations in loading of NiR/CHIT-V mixture and/or in uniformity of drop-coated HPU membrane with the manual operation.

An interference study showed that common anions found in water samples such as chlorate, chloride, sulfite and sulfate didn’t interfere with the detection. However, nitrate interfered strongly with the detection: the sensitivity of the sensor for this anion was as high as 9.6 nA/μM, which is 64% of that for nitrite. This interference effect should be attributed to the inherent character of the Cu-NiR used in this study because control experiments with a co-immobilized BSA and CHIT-V electrode (no NiR, data not shown) did not show a response to nitrate. It was reported that the radical cation of methyl viologen is not able to reduce nitrate [49]. In real samples, nitrite and nitrate are commonly found together [3], therefore, direct and selective determination of nitrite in real samples with the biosensor reported here seems difficult at present. However, total determination of nitrite and nitrate seems possible.

The stability of the sensor (*i.e.* retaining 80% of initial activity) was ∼65 days under ambient air at room temperature storage conditions. This long-term storage stability of the sensor is superior to those reported previously [12,13,15,18]. In a similar case of co-immobilization of Cu-NiR (the same enzyme) and PAPS-V by drop-coating of HPU, the storage stability (under the same conditions) was 51 days [22], and in another case of co-immobilization of Cu-NiR and methyl viologen by poly(vinyl alcohol) (PVA) entrapment, the storage stability was 24 days [17]. The favorable interaction between Cu-NiR and chitosan, as discussed in the *K*_M_^app^ section, may be responsible for the prolonged stability of the sensor reported here, besides the durability of the enzyme itself [36,37]. It was also reported that chitosan is able to increase the stability and activity of immobilized enzymes [43].

Based on the data reported here, it can be anticipated that a sensitive biosensor with durability, rapidity, reproducibility, selectivity and simplicity may be used for determination of nitrite in real samples in the near future.

## 4. Conclusions

Cu-NiR and CHIT-V were co-immobilized on a GCE surface by drop-coating of HPU. The electrode showed high catalytic effect for the electro-enzymatic reduction of nitrite. Under the optimized conditions, the NiR and CHIT-V co-immobilized biosensor showed a detection limit of 40 nM and a long-term storage stability of 65 days, which imply that this type of biosensor has the potential to be used in practical applications. In spite of these advantages this type biosensor suffered a strong interference effect from nitrate, a ubiquitous anion in real water samples. Although this biosensor can ot be directly used in real samples at present, a flow injection analysis (FIA) system consisting of dual-working electrodes may be used in simultaneous detection of nitrite and nitrate.
